# Elevated desmoglein‐2 expression in multiple myeloma is a prognostic marker across genomic subtypes with impact on high‐risk cytogenetics and a distinct gene expression profile

**DOI:** 10.1111/bjh.70554

**Published:** 2026-05-18

**Authors:** Barbara J. McClure, John Toubia, Charlotte E. J. Toomes, O. Giles Best, Angelina Yong, Cindy H. Lee, Claudine S. Bonder, Chung Hoow Kok

**Affiliations:** ^1^ Centre for Cancer Biology, College of Health Adelaide University and SA Pathology Adelaide South Australia Australia; ^2^ Data and Bioinformatics Innovation, Department of Genetics and Molecular Pathology SA Pathology Adelaide South Australia Australia; ^3^ Flinders Health and Medical Research Institute, College of Medicine and Public Health, Flinders University Bedford Park South Australia Australia; ^4^ Department of Haematology Royal Adelaide Hospital, Central Adelaide Local Health Network Adelaide South Australia Australia; ^5^ School of Medicine College of Health, Adelaide University Adelaide South Australia Australia

**Keywords:** desmoglein‐2, multiple myeloma, prognostic biomarker

To the Editor,

Multiple myeloma (MM) is an incurable malignancy, primarily of the bone marrow (BM) caused by uncontrolled proliferation of neoplastic antibody‐secreting plasma cells (PCs). It is the second most common haematological malignancy with >188 000 new cases diagnosed annually worldwide.^1^ Despite therapy advances, the progression‐free survival (PFS) for MM patients over the last two decades has not improved and the 5‐year overall survival (OS) has improved but remains poor at 54%.^1^, ^2^ Furthermore, the recent introduction of therapeutic risk stratification based on high‐risk genomic lesions^3^ has not led to an improvement in OS.^2^, ^4^, ^5^ Rapid identification of high‐risk patients at diagnosis would undoubtedly improve outcomes by informing early treatment selection.

The presence of cytogenetic lesions, namely t(4;14), t(14;16) and deletion of 17p (del17p), are considered indicators of high‐risk disease^3^ and are now incorporated into the Revised International Staging System (R‐ISS), the most commonly implemented MM risk stratification strategy. This means that identification of these lesions is crucial prior to treatment initiation. Recently, 1q21 gain/amplification (1q+), mutations of *TP53* and biallelic deletion of 1p32 (del1p) have also been identified to be prognostic for high‐risk disease. However, some patients with standard cytogenetics can do poorly, suggesting the presence of additional biological or clinical factors that influence disease trajectory. Identifying these factors is critical to refining risk stratification and improving effective personalised therapeutic strategies in MM.

We previously identified that desmoglein‐2 (*DSG2*) expression is significantly elevated in the PCs of approximately 30% of MM patients and is prognostic for a fourfold increased risk of death.^6^ DSG2 is a cell surface expressed cadherin family adhesion protein that plays non‐canonical roles in supporting proliferation and survival of non‐desmosome‐forming progenitor cells (endothelial, haematopoietic)^7^, ^8^ and has been increasingly implicated in solid tumour progression. The aim of this study was to elucidate the capacity of *DSG2* expression as a prognostic marker in newly diagnosed (ND) MM within clinically defined cytogenetic cohorts for the first time and to explore the *DSG2*‐associated gene expression profile that may contribute to disease progression.

The level of *DSG2* expression and its prognostic value were determined within contemporary cytogenetic subtypes using publicly available transcriptomic data from CD138+ malignant PCs from 678 NDMM patients (where cytogenetic/FISH and clinical outcome data were available) (Table S1), sourced from the MM research fund (MMRF)‐coMMpass cohort (NCT01454297) (MMRF gateway). In this cohort, samples were stratified into quartiles based on *DSG2* expression; MM patients within the top 25% for *DSG2* (Q4) expression had significantly shorter PFS (median survival 27.8 vs. 37.2 months, HR 1.42, 95% CI 1.12–1.78, *p* = 0.026) (Figure 1A) and OS times (54.9 months vs. not reached, HR 1.81, 95% CI 1.37–2.41, *p* < 0.0001) (Figure 1B) compared to patients within the lowest 25% (Q1). A multivariable analysis (MVA) was performed by including age, gender, ISS, SCT (stem cell transplant), treatment and *DSG2*. We identified that *DSG2* remains statistically significant as independent predictor for PFS (*p* = 0.007) and OS (*p* < 0.001), after adjusting for covariables (Figure S1A,B). This result is consistent with our previous report.^6^ The reduced survival associated with elevated *DSG2* is independent of haematopoietic stem cell transplantation (Figure S1C,D).

**FIGURE 1:**
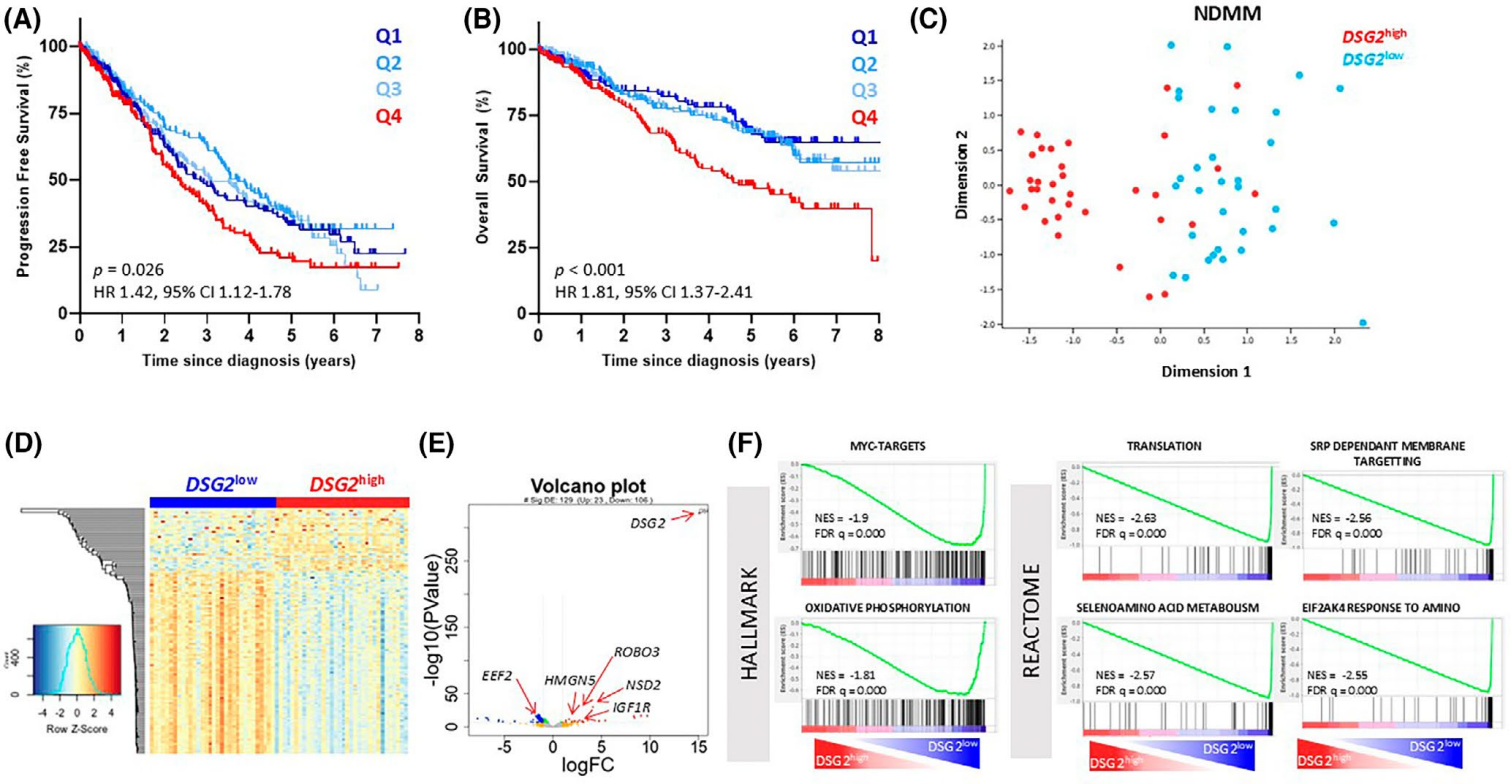
Kaplan–Meier analyses of (A) progression‐free survival and (B) overall survival among newly diagnosed multiple myeloma (NDMM) patients. Patients were stratified into quartiles (Q1–Q4, lowest to highest) based on *DSG2* expression. Data were obtained from the multiple myeloma research fund (MMRF)‐coMMpass‐gateway (*n* = 678). *DSG2* expression was assessed in CD138+ plasma cells and survival was compared using a log‐rank test for Q4 versus Q1/2/3 combined. Q1 *n* = 170, Q2, *n* = 170, Q3 *n* = 169 and Q4 *n* = 169. (C) Patients were stratified into cohorts of the highest and lowest 5% based on their *DSG2* expression in PCs from NDMM patients, *DSG2* ^high^ (red, *n* = 34) and *DSG2* ^low^ (blue, *n* = 33) respectively. Groups were assessed by supervised clustering using multidimensional scaling (MDS) showing dimensions 1 and 2, to visualise differences in global gene expression between the groups. (D) Heat map showing the 129 significantly differentially expressed genes between groups (FDR < 0.05, FC > |2|). (E) Volcano plot showing the degree of change versus significance. Each dot represents one gene with red and blue circles indicating significantly up‐ and downregulated genes respectively (FDR < 0.05, FC > |2|). (F) Gene set enrichment (GSEA) profiling of differential gene expression between *DSG2* ^high^ (*n* = 34) and *DSG2* ^low^ (*n* = 33) in PCs from NDMM patients in the MMRF‐coMMpass‐gateway dataset. GSEA plots detailing the pathways identified from the Hallmark and Reactome gene sets using a FDR of *q* < 0.05.

To elucidate if elevated *DSG2* expression is linked to altered gene expression modules, we next performed a differential gene analysis using the TRANSECT application for assessment of altered gene regulation^9^ using the application specific recommended number of samples per group (stratum) for highest and lowest *DSG2* expression, *DSG2* ^low^ (*n* = 33) and *DSG2* ^high^ (*n* = 34) (Supporting Information Methods and Table S2A,B), and importantly, a continuous variable demonstrated that the resulting signature did not change (data not shown). Multidimensional scale (MDS) analysis revealed a distinct gene expression profile in *DSG2* ^high^ versus *DSG2* ^low^ NDMM (Figure 1C), with 129 differentially expressed genes identified (false discovery rate [FDR] < 0.05, FC > |2|), including 23 upregulated and 106 downregulated genes (Figure 1D; Table S3). Within the *DSG2* ^high^ cohort, upregulation of *NSD2*, *KLF4*, *IGF1R*, *ROBO3* and *HMGN5* (all FDR < 0.001) and downregulation of *EEF2* and multiple ribosomal protein large/small subunit (*RPL*/*RPS*) genes (all FDR < 0.001) were observed compared to *DSG2* ^low^ samples (Figure 1E; Table S3). Interestingly, upregulation of *HMGN5* has previously been associated with a poor prognosis gene signature in MM.^10^ While *ROBO1* has been reported to support homing and dissemination of MM‐PCs within the BM niche,^11^ and elevated levels of *ROBO3* occur in t(4;14) MM.^12^ Gene set enrichment analysis (GSEA) showed that elevated *DSG2* expression was associated with a downregulation of genes involved in pathways related to *MYC* targets and genes involved in oxidative phosphorylation, ribosomal, amino acid and selenoamino acid metabolism and translation pathways (FDR *q* < 0.001) (Figure 1F; Figure S2; Table S4). Interestingly, selenium and its metabolism have been shown to significantly improve the immune response in MM by regulating the cross‐talk between tumour cells and immune cells and by reshaping the tumour microenvironment.^13^

*DSG2* expression within the primary cytogenetic subsets was assessed on CD138+ MM‐PCs (*n* = 678) from NDMM patients using data from the MMRF‐coMMpass dataset. This included patients with t(4;14) (12.7%, *n* = 86), t(6;14) (1.5%, *n* = 10), t(11;14) (19.6%, *n* = 133), t(14;16) (3.8%, *n* = 26) and t(14;20) (1.5%, *n* = 10) (Figure 2A). *DSG2* expression was elevated within PCs from NDMM patients with t(4;14) (88.4%), followed by t(14;20) (30%), t(11;14) (23.3%) or t(14;16) (15.4%) (Figure S3A). The most common secondary genomic lesion was hyperdiploid (58.6%, *n* = 397) followed by 1q+(38.6%, *n* = 262), del1p (25.1% *n* = 170), del17/17p (13.4%, *n* = 91), p53 mutation (10.0%, *n* = 68); within all these subgroups of patients, bimodal expression *DSG2* expression was observed (Figure 2B).

**FIGURE 2:**
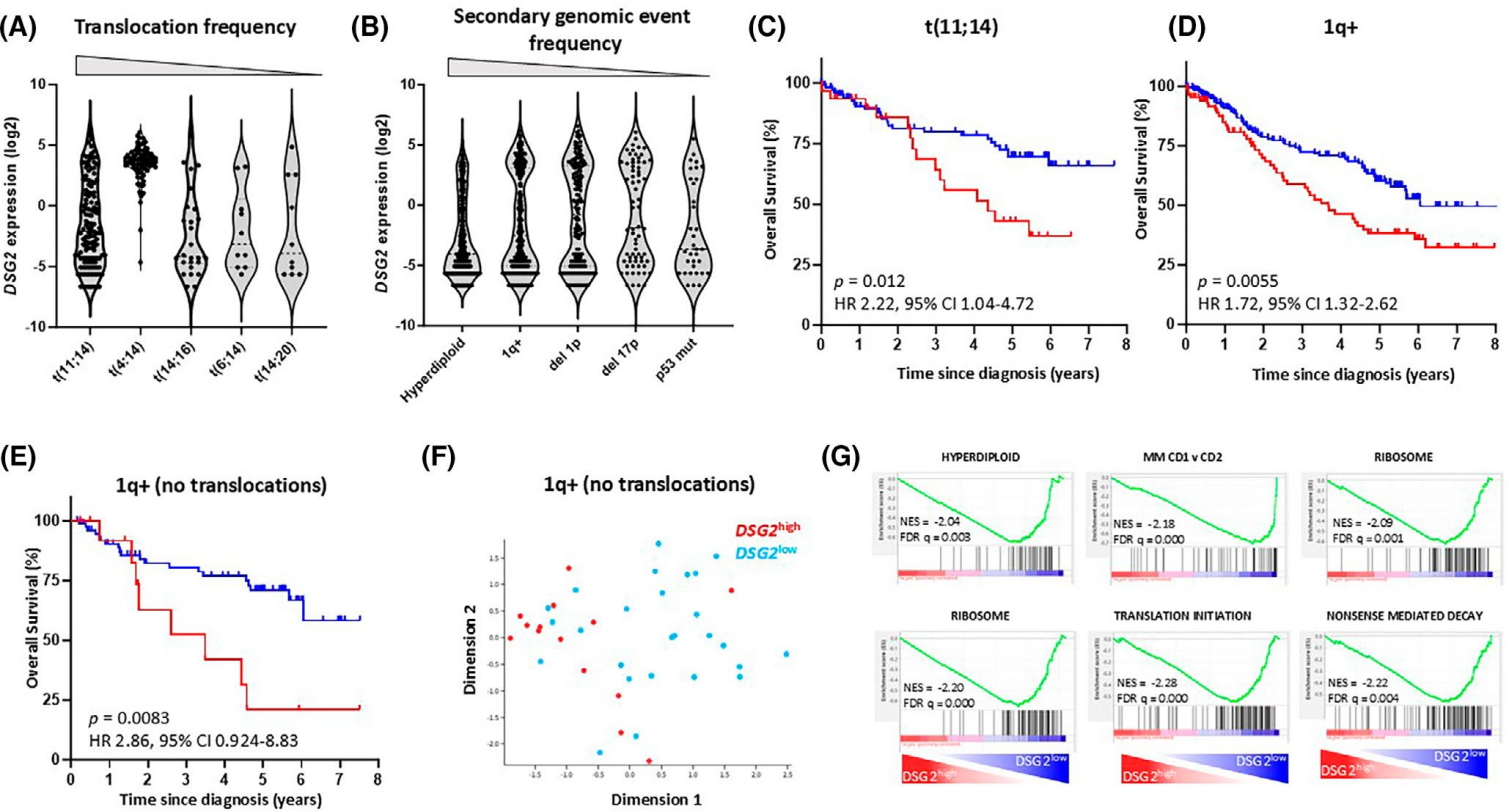
(A) Expression of *DSG2* compared between PCs from NDMM patients in each of the cytogenetic subgroups shown, according to the primary translocation present. (B) Expression of *DSG2* in PCs from NDMM patients was compared following grouping of the patients according to their secondary genomic lesions. (C) Kaplan–Meier analysis of percent overall survival (OS) was assessed for NDMM patients with t(11;14) stratified as either *DSG2* ^high^ (red, top 25%) or *DSG2* ^low^ (blue, bottom 75%) based on expression in CD138+ PCs from the multiple myeloma research fund (MMRF)‐coMMpass dataset (*n* = 31 and *n* = 104 respectively). *DSG2* ^high^ patients were defined as those within the top quartile (Q4) of *DSG2* expression. Survival functions were compared using a log‐rank test. (D) Kaplan–Meier analysis of overall survival (OS) was assessed for 1q+ NDMM patients grouped as either *DSG2* ^high^ (red) or *DSG2* ^low^ (blue) according to *DSG2* expression in CD138+ PCs; data analysed were from the MMRF‐coMMpass dataset (*n* = 84 and *n* = 178 for *DSG2* ^high^ and *DSG2* ^low^ respectively). *DSG2* ^high^ patients were defined as those within the top quartile of *DSG2* expression. Survival functions were compared using a log‐rank test. (E) A subset of 1q+ NDMM patients who did not harbour any known translocations were stratified into *DSG2* ^high^ and *DSG2* ^low^ (*n* = 14 and *n* = 76, respectively) and Kaplan–Meier analysis performed. (F) NDMM 1q+ (with no translocations) samples grouped as *DSG2* ^high^ (red, *n* = 14) or *DSG2* ^low^ (blue, *n* = 26) were assessed by supervised clustering using multidimensional scaling (MDS), showing dimensions 1 and 2, to visualise differences in global gene expression between the groups. (G) Gene set enrichment (GSEA) profiling showing differential gene expression in PCs from *DSG2* ^high^ (*n* = 14) and *DSG2* ^low^ (*n* = 26), 1q+ (no translocations) NDMM patients from the MMRF‐coMMpass dataset. GSEA plots detailing the pathways identified obtained from the Hallmark and Reactome gene sets, with an FDR of *q* < 0.05.

We next investigated NDMM patients harbouring specific primary translocations (derived by FISH analysis and where sufficient samples were available) for Kaplan–Meier analyses in an exploratory way to understand the impact of high *DSG2* expression within cytogenetic subtypes. These included translocations associated with standard risk, t(11;14), and high‐risk disease, t(4;14) and t(14;16).^14^ PFS and OS were assessed following stratification based on *DSG2* expression into the top 25% (*DSG2* ^high^) and remaining 75% (*DSG2* ^low^). Importantly, in NDMM patients harbouring the standard risk t(11;14) translocation (*n* = 133), elevated *DSG2* expression was associated with significantly worse OS (56.6 months vs. undefined, HR 2.22, 95% CI 1.04–4.72, *p* = 0.012) (Figure 2C) and PFS (48.1 months vs. undefined, HR 1.92, 95% CI 0.928–3.98, *p* = 0.0416) (Figure S3B) compared to those with low *DSG2* expression. In the cohort of patients with known high‐risk translocations t(4;14) (*n* = 87) and t(14;16) (*n* = 27), *DSG2* levels did not correlate with either PFS (Figure S3C,D) or OS (Figure S3E,F).

Patients with 1q+ alterations and *DSG2* ^high^ expression had worse OS compared to 1q+ patients with *DSG2* ^low^ expression, (44.1 vs. 72.6 months, HR 1.72, 95% CI 1.32–2.62, *p* = 0.0055) (Figure 2D) and significantly inferior PFS (20.5 vs. 35.2 months, HR 1.78, 95% CI 1.24–2.54, *p* = 0.0004) (Figure S4A). No significant difference in OS or PFS within the patients with del1p, del17/17p or p53 mutation subgroups was observed between patients with high and low *DSG2* expression (Figure S4A,B). Next, we evaluated baseline clinical variables to identify any significant association with 1q+ *DSG2* ^high^ versus *DSG2* ^low^ patients (*n* = 91). We identified t(4;14), del1p and hyperdiploid (*p* < 0.001) as significant factors associated with *DSG2* ^high^ versus *DSG2* ^low^ 1q+NDMM (Table S5) and that incorporating *DSG2* level showed improved performance than t(11;14) or 1q+ alone (Figure S4C).

To identify pathways associated with elevated *DSG2*, we interrogated the t(11;14) cohort and assessed only samples that lacked co‐existing 1q+ high‐risk alterations (*n* = 102), which could influence prognosis. In this t(11;14)/without 1q+ cohort, *DSG2* remained prognostic for OS (48.9 months vs. undefined, HR 2.57, 95% CI 0.976–6.78, *p* = 0.0125) (Figure S5A) but not PFS (38.7 months vs. 50.0 months, *p* = 0.4238) (Figure S5B). MDS analysis did not reveal a distinct gene expression profile in *DSG2* ^high^ versus *DSG2* ^low^ NDMM (Figure S5C,D), with only seven genes identified as being significantly differentially regulated (data not shown).

Given the poor prognostic impact of t(4;14), we investigated survival and differential gene expression on 1q+ patients who lacked t(4;14) to clarify the impact of *DSG2* in isolation. Patients whose MM‐PCs had 1q+ alterations (no translocations) and *DSG2* ^high^ expression had worse OS compared to patients with *DSG2* ^low^ expression (41.8 vs. undefined, HR 2.86, 95% CI 0.924–8.83, *p* = 0.0083) (Figure 2E) but not PFS (21.4 vs. 42.3 months, *p* = 0.1178) (Figure S5E). MDS analysis of *DSG2* ^high^ (*n* = 25) and *DSG2* ^low^ (*n* = 25) NDMM‐PCs from patients with 1q+ (no translocation present) (Table S6) identified six differentially expressed genes (FDR < 0.05, FC > |2|) that were all upregulated in the *DSG2* ^*high*^ patient samples (Figure 2F; Figure S5F; Table S7). GSEA showed that elevated *DSG2* expression in 1q+ (no translocations) NDMM‐PCs was associated with downregulation of genes in pathways related to ribosomes, nonsense‐mediated decay and translation (FDR *q* < 0.05) (Figure 2G; Figure S5G; Table S8). Downregulation of genes associated with ribosomal pathways has previously been reported to contribute to suboptimal response to bortezomib.^15^ Given the poor response rates of *DSG2* ^high^ NDMM patients to this proteasome inhibitor,^6^ the influence of ribosomal pathways warrants further investigation to elucidate the role(s) of *DSG2* in high‐risk MM.

This study highlights the potential of *DSG2* as a novel prognostic biomarker for high‐risk NDMM, reveals that *DSG2* expression is associated with chromosomal aberrations and identifies pathways associated with increased *DSG2*. Elevated *DSG2* expression was predictive of poor OS among NDMM patients, including those with t(11;14) and/or 1q+, regardless of the presence of additional genomic alterations. Elevated *DSG2* expression was associated with a distinct gene expression profile in NDMM‐PCs and within patients with the 1q+ high‐risk cytogenetic lesion. These findings implicate *DSG2* as an underappreciated MM prognostic biomarker and provide the rationale for further investigation into its role(s) in MM, including whether targeting DSG2 may represent a novel approach for precision medicines. Moreover, this study suggests that DSG2 expression analysis would complement the current risk stratification strategies, aiding in the rapid identification of high‐risk patients at diagnosis.

## AUTHOR CONTRIBUTIONS

**Claudine S. Bonder:** Investigation; writing – review and editing; supervision; funding acquisition. **John Toubia:** Investigation; writing – review and editing; methodology; formal analysis; data curation. **Chung Hoow Kok:** Conceptualization; investigation; writing – review and editing; supervision; data curation; formal analysis. **Barbara J. McClure:** Conceptualization; writing – original draft; formal analysis; investigation; data curation. **Angelina Yong:** Investigation; writing – review and editing. **O. Giles Best:** Writing – review and editing; investigation. **Cindy H. Lee:** Investigation; writing – review and editing. **Charlotte E. J. Toomes:** Writing – review and editing; investigation.

## FUNDING INFORMATION

This work was supported in part by funding to CSB from the National Health and Medical Research Council (NHMRC) (GNT2013460) and to BJM from the University of South Australia.

## CONFLICT OF INTEREST STATEMENT

CSB has received research funding from Carina Biotech Pty. CL is an Advisory Board member for Janssen, Takeda, Pfizer, Antengene and BMS and has received research funding from them. All authors declare no competing interests.

## ETHICS STATEMENT

This study analysed publicly available, de‐identified datasets and did not involve the recruitment of new human subjects. Therefore, ethical approval was not required.

## PATIENT CONSENT STATEMENT

Patients in the MMRF CoMMpass study (NCT01454297) provided written consent to participate in a longitudinal, clinical‐genomic study. The consent was conducted as per the Declaration of Helsinki guidelines.

## Supporting information


Figures S1–S5.

Tables S1–S8.

Data S1.

## Data Availability

These data were generated as part of the Multiple Myeloma Research Foundation Personalized Medicine Initiatives (https://research.themmrf.org and www.themmrf.org). Data were retrieved from the Genomic Data Commons (GDC) data portal on 28 July 2026 (https://portal.gdc.cancer.gov/).
