# Supplementary material for: Elevated desmoglein‐2 expression in multiple myeloma is a prognostic marker across genomic subtypes with impact on high‐risk cytogenetics and a distinct gene expression profile
Source: Br J Haematol. 2026 May 18;209(1):306–10. doi: 10.1111/bjh.70554 (PMC13340476; doi:10.1111/bjh.70554)

Supplementary Figure 1

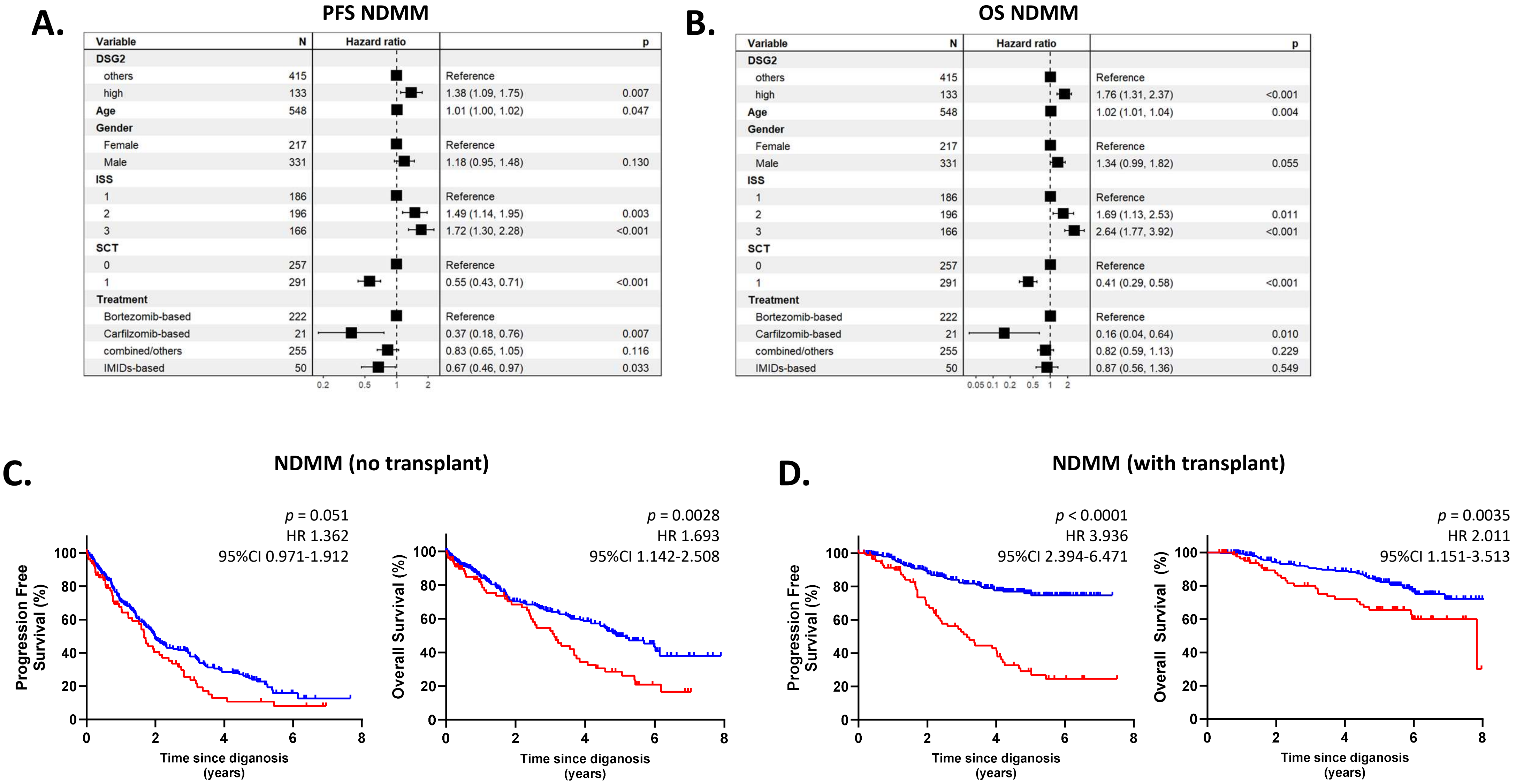

Supplementary Figure 2

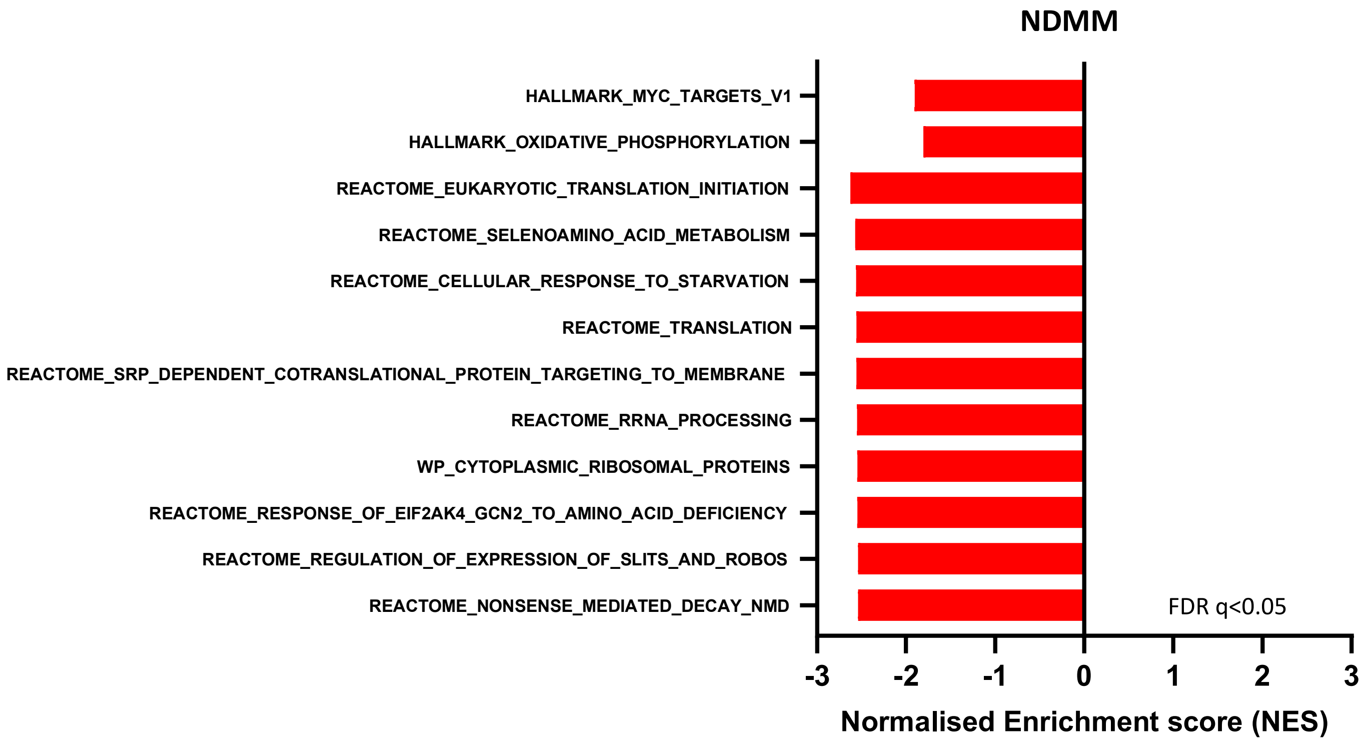

Supplementary Figure 3

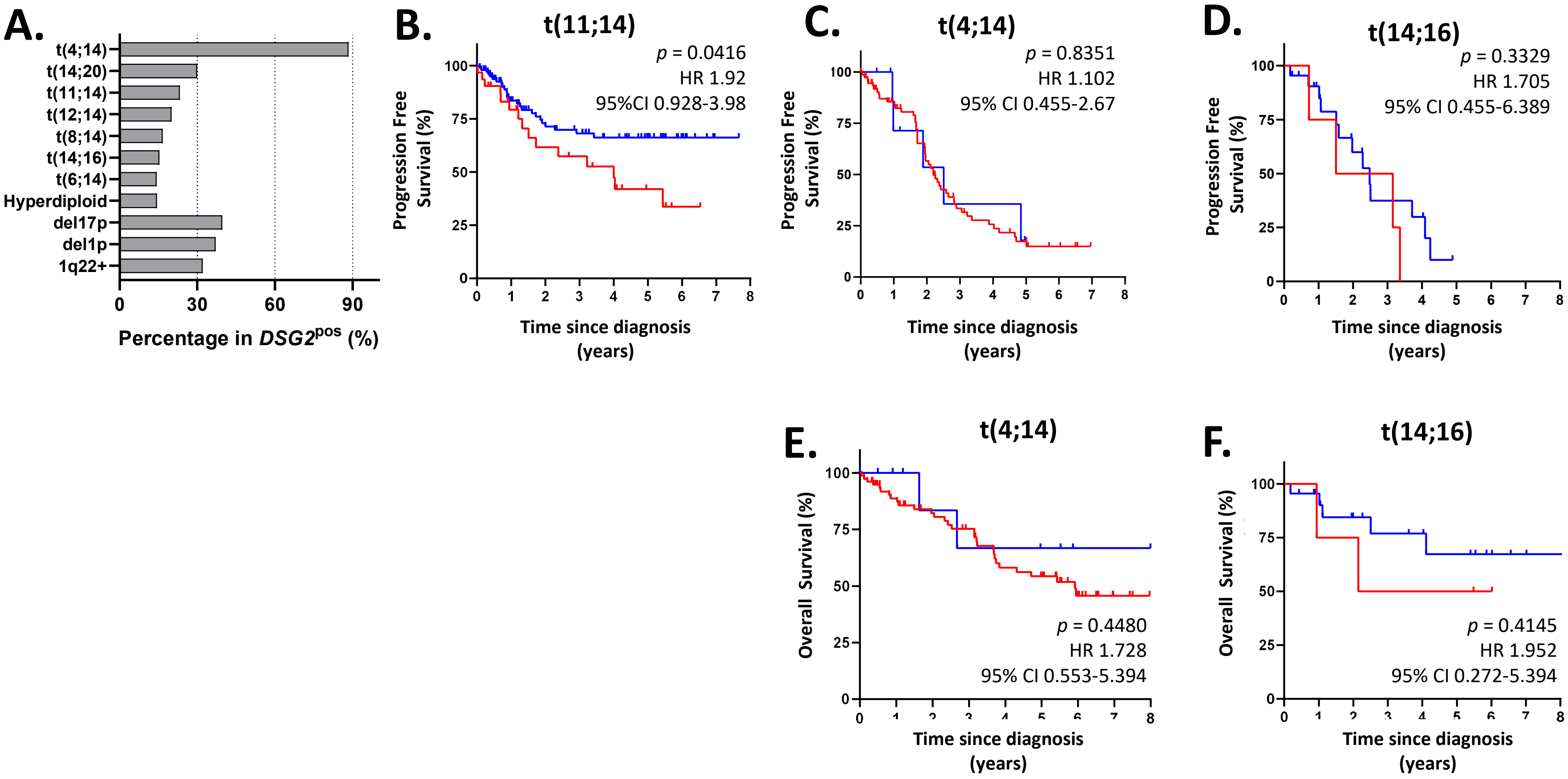

Supplementary Figure 4

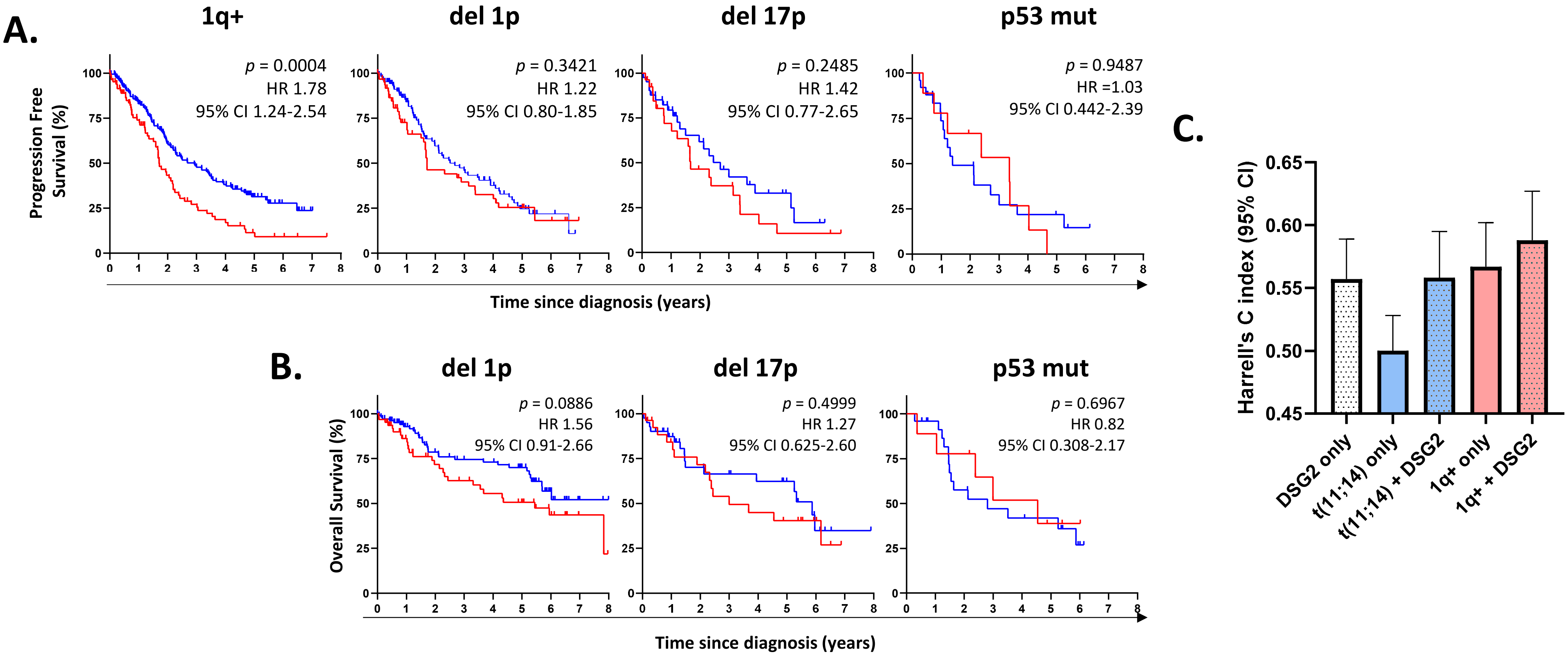

Supplementary Figure 5

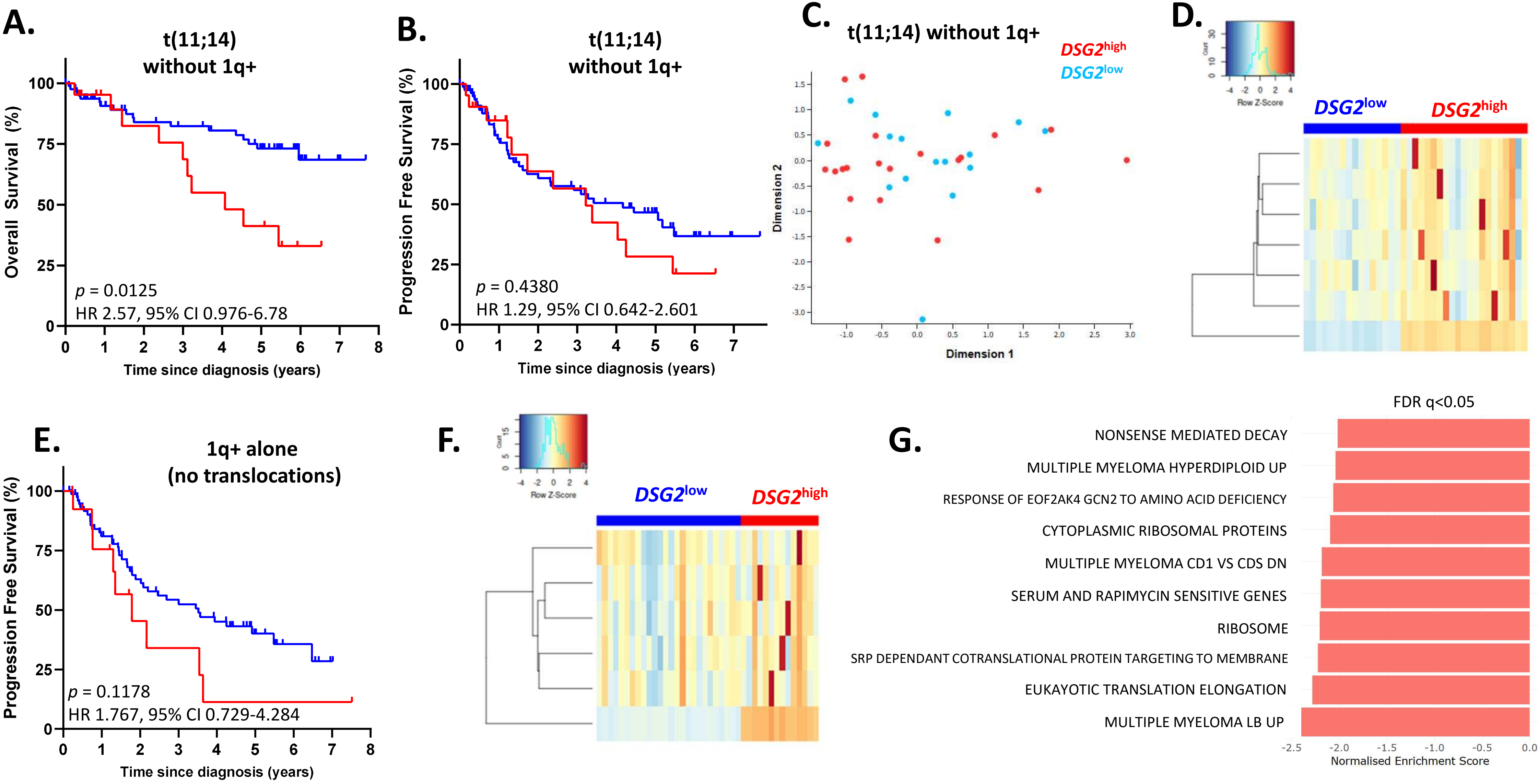

Supplement: Supplementary file 1 — Figures S1–S5. [file BJH-209-306-s003.pdf]
