# Supplementary material for: Elevated desmoglein‐2 expression in multiple myeloma is a prognostic marker across genomic subtypes with impact on high‐risk cytogenetics and a distinct gene expression profile
Source: Br J Haematol. 2026 May 18;209(1):306–10. doi: 10.1111/bjh.70554 (PMC13340476; doi:10.1111/bjh.70554)
Supplement: Supplementary file 3 — Data S1. [file BJH-209-306-s002.pdf]

Supporting Information for:

McClure et al.

Elevated desmoglein-2 expression in multiple myeloma is a prognostic marker across genomic subtypes with impact on high-risk cytogenetics and a distinct gene expression profile

1. Supplementary Methods

2. Supplementary References

3. Supplementary Figures 1-4

4. Supplementary Tables S1-S8

Supplementary Methods

Data sets

Uniformly derived gene expression measurements on bone marrow CD138+ plasma cells for each of the 678 newly diagnosed multiple myeloma (NDMM) patients where cytogenetic/FISH data was available were retrieved from the Genomic Data Commons (GDC) data portal (https://portal.gdc.cancer.gov/) and tabulated. The provided expression measurements for each patient in the cohort were used as is without additional batch correction. The clinical data (seqFISH, pairoscope result, somatic mutation SNV/INDEL, PFS and OS) were obtained from the MMRF CoMMpass gateway (IA18). The cytogenetic assignment for each patient available from the coMMpass data set was determined by FISHseq (>20%). As such 1q+ and 1q amp cannot be distinguished and for this study have been considered as a group termed 1q+. Similarly, del 17 and del 17p, which are not distinguished by FISHseq, are also considered as a group for this study and demoted del 17p. For alterations impacting *TP53* samples with either *TP53* mutations or deletions were grouped together due to their small individual sample sizes to permit Kaplan-Meier analysis.

DSG2 expression data (*n*=678) (TPM range: 0.01 – 95.31, average=4.12) was used to stratify NDMM into *DSG2*^{high} and *DSG2*^{low} groups based on high (highest 5% *DSG2* expression; n=34; average TPM=37) and low (lowest 5% *DSG2* expression; n=33; average TPM=0.010) thresholds respectively. *DSG2* expression data specifically for 1q+ (no translocation) patients (*n*=102) (TPM range: 0.01 – 40.55, average=4.74) was used to stratify patients into *DSG2*^{high} and *DSG2*^{low} groups based on high (highest 25% *DSG2* expression; n=14; average TPM=13.53) and low (lowest 25% *DSG2* expression; n=26; average TPM =0.19) thresholds respectively.

Differential gene expression and Gene set enrichment analysis

Cohort stratification and the subsequent global gene expression and gene set enrichment analyses was performed using the Transcriptome Analysis of Stratified Cohorts (TRANSECT) command line application, adopting the application specific recommended settings (1). Briefly; differential expression analysis between the two resulting groups was evaluated from TMM normalized gene counts using R (version 4.4.2) and edgeR (version 4.0.16) (2) following protocols as described (1). Genes found to be expressed at low levels (count per million, CPM<5) in more than the smallest group size were filtered prior to conducting differential expression tests. Graphical representations of differentially expressed genes were generated using Glimma (3). The Gene Set Enrichment Analysis software package (GSEA v4.1.0) was used to look for coordinate expression to groups of genes in the Molecular Signatures Database (MSigDB v7.1) (4, 5). Genes were ranked for the GSEA analysis (GSEAPreranked) by calculating the “directional” negative log FDR (sign of fold change * -log10(FDR)).

Statistical Analysis

Median (interquartile range, IQR) and frequencies (percentage) were used for continuous and categorical variables, respectively. Categorical variables were compared using Chi-squared test and continuous variables using Mann-Whitney test. Survival probabilities over time were estimated using Kaplan-Meier, and comparisons of survival across subgroups were conducted using the two-sided log-

rank test. patients were stratified into cohorts based on DSG2 expression of the top 25% (*DSG2*^{high}) and remaining 75% (*DSG2*^{low}) and analysed for PFS and OS. Univariate analysis for OS and PFS were performed with Log-rank test. Multivariable analysis was performed using Cox proportional hazards model of OS and PFS. Hazard ratios and 95% confidence interval (CI) were reported for covariates, along with *p* values from the Wald test. *p* values <0.05 were considered statistically significant. All statistical analyses were conducted using the R statistical platform (https://www.r-project.org/) v.4.1.1. and GraphPad PRISM version 10.

Supplementary References

1. Toubia J, Kusay Y, Maqsood M, Warnock NI, Lawrence DM, Bracken CP, et al. TRanscriptome ANalysis of StratifiEd CohorTs (TRANSECT) enables automated assessment of global gene regulation linked to disparate expression in user defined genes and gene sets. NAR Genom Bioinform. 2025;7(2):lqaf041.
2. Robinson MD, McCarthy DJ, Smyth GK. edgeR: a Bioconductor package for differential expression analysis of digital gene expression data. Bioinformatics. 2010;26(1):139-40.
3. Su S, Law CW, Ah-Cann C, Asselin-Labat ML, Blewitt ME, Ritchie ME. Glimma: interactive graphics for gene expression analysis. Bioinformatics. 2017;33(13):2050-2.
4. Mootha VK, Lindgren CM, Eriksson KF, Subramanian A, Sihag S, Lehar J, et al. PGC-1alpha-responsive genes involved in oxidative phosphorylation are coordinately downregulated in human diabetes. Nat Genet. 2003;34(3):267-73.
5. Subramanian A, Tamayo P, Mootha VK, Mukherjee S, Ebert BL, Gillette MA, et al. Gene set enrichment analysis: a knowledge-based approach for interpreting genome-wide expression profiles. Proc Natl Acad Sci U S A. 2005;102(43):15545-50.

Supplementary Figure 1. Adjusted hazard ratios of *DSG2* expression and subgroup variables (age, gender, ISS, SCT, treatment and *DSG2*) calculated by multivariate Cox regression for **(A)** progression free survival (PFS) and **(B)** overall survival (OS). Kaplan-Meier analyses of progression free survival and overall survival among newly diagnosed MM (NDMM) patients that **(C)** did not undergo stem cell transplant or **(D)** received a stem cell transplant. Data were obtained from the MMRF-coMMpass-gateway (*n*=319). Patients were stratified into *DSG2*^{high} (Q4 red, top 25%, n=80) or *DSG2*^{low} (Q1-3 blue, bottom 75%, n=239). *DSG2* expression was assessed in CD138+ plasma cells and survival was compared using a log rank test.

Supplementary Figure 2. Gene set enrichment profiling of transcriptomic sequencing data of *DSG2*^{high} (*n*=33) versus *DSG2*^{low} (*n*=33) (top and bottom 5% of *DSG2* expression) from NDMM CD138+MM-PCs FDR $q<0.05$.

Supplementary Figure 3. **(A)** Percentage of patients with *DSG2*^{high} expression (Q4) in combination with primary and secondary genomic alterations. Cytogenetic subgroups t(11;14), t(4;14) and t(14;16) were analysed by Kaplan-Meier analysis for **(B, C, D)** percent progression free survival and **(E, F)** overall survival of newly diagnosed MM (NDMM) patients. Patients were stratified into quartiles (Q1 – Q4, lowest to highest of *DSG2* expression). Data were obtained from the MMRF-coMMpass-gateway (t(11;14) n= 133, t(4;14) n= 87, t(14;16) n= 27). *DSG2* expression determined from CD138+ plasma cells and survival was compared using a log rank test.

Supplementary Figure 4. Cytogenetic subgroups were analysed by Kaplan-Meier analysis for percent **(A)** progression free survival and **(B)** overall survival and stratified into *DSG2*^{high} (**red**, top 25%) or *DSG2*^{low} (**blue**, bottom 75%) expression from the MMRF-coMMpass data set (1q+ n= 262, del 1p n= 170, del 17p n= 91, p53mut = 34). *DSG2* expression determined from CD138+ NDMM plasma cells and survival was compared using a log rank test. **(C)** Improved confidence interval indicates DSG2 complements current risk.

Supplementary Figure 5. Kaplan-Meier analysis of percent of **(A)** OS or **(B)** PFS of NDMM patients classified as either *DSG2*^{high} (**red**) or *DSG2*^{low} (**blue**) according to the expression on CD138+ MM plasma cells with t(11;14) and lacking 1q+ from the MMRF-coMMpass data set (*n*=102). **(C)** The t(11;14) without 1q+ subset were stratified into *DSG2*^{high} (**red**) and *DSG2*^{neg} (**blue**) groups and were assessed by supervised clustering using multidimensional scaling (MDS) plots of log-CPM values and dimensions 1 and 2, to identify differences in gene expression. **(D)** Heatmap showing the seven genes which were significantly differentially expressed based on an FDR of $p<0.05$ **(E)** Kaplan-Meier analysis of percent of progression free survival (PFS) of NDMM patients classified as either *DSG2*^{high} (**red**) or *DSG2*^{low} (**blue**) according to the expression on CD138+ MM plasma cells for patients with 1q+ (and no other translocations), from the MMRF-coMMpass data set (*n*=91). **(F)** Heatmap showing the six genes which were significantly differentially expressed based on an FDR of $p<0.05$. **(G)** Gene set enrichment profiling of transcriptomic sequencing data from *DSG2*^{high} (*n*=22) versus *DSG2*^{low} (*n*=69) 1q+ (and no other translocations) NDMM-PCs FDR $q<0.05$.
